# The use of computed tomography during follow-up after ablation of cT1 renal cell carcinoma: evidence for overuse

**DOI:** 10.1007/s00330-026-12345-6

**Published:** 2026-02-06

**Authors:** Marlin A. A. Reijerink, Luna van den Brink, Michael M. E. L. Henderickx, Otto M. van Delden, Harrie P. Beerlage, Axel Bex, Faridi S. Jamaludin, Mitra Nekouei Shahraki, Patricia J. Zondervan, Jaap Stoker

**Affiliations:** 1 Amsterdam UMC, Location University of Amsterdam, Department of Radiology and Nuclear Medicine, Meibergdreef 9, Amsterdam, The Netherlands; 2 Cancer Center Amsterdam, Imaging and Biomarkers, Amsterdam, The Netherlands; 3 Amsterdam UMC, Location University of Amsterdam, Department of Urology, Meibergdreef 9, Amsterdam, The Netherlands; 4 Netherlands Cancer Institute (NKI), Department of Urology, Plesmanlaan 121, Amsterdam, The Netherlands; 5 Royal Free Hospital, Department of Urology, 10 Pond Street, London, United Kingdom; 6 Amsterdam UMC, Location University of Amsterdam, Medical Library AMC, Meibergdreef 9, Amsterdam, The Netherlands; 7 Amsterdam UMC, Location University of Amsterdam, Department of Epidemiology and Data Science, Meibergdreef 9, Amsterdam, The Netherlands

**Keywords:** Computed tomography, Follow-up, Recurrences

## Abstract

****Objective**:**

This systematic review aims to assess whether studies that followed the 2016 and updated 2024 European Association of Urology (EAU) Renal Cell Carcinoma (RCC) guidelines for CT during follow-up after tumor ablation (TA) yield different oncological outcomes compared to studies that performed more frequent imaging.

****Materials and methods**:**

A literature search of relevant search engines was performed up to June 6th, 2025. Studies that reported follow-up schedules of patients after TA for cT1 RCC were included. Studies utilizing more CT scans than recommended by the 2016 and 2024 EAU guidelines were compared with those adhering to the guidelines. Data on recurrences and survival were analyzed.

****Results**:**

Thirty-seven studies met the inclusion criteria, involving patients with cT1 RCC treated with TA. The mean 5-year overall survival rate was 82.9%. The pooled recurrence rate was 7.7% in studies that performed more imaging than recommended by the 2016 EAU guideline, compared with 12.3% in studies that adhered to the guideline (*p* = 0.19). All studies performed more imaging than recommended by the updated 2024 guidelines. Risk of bias was moderate to high in most studies.

****Conclusion**:**

The majority of included studies conducted more frequent imaging than advised by the 2016 EAU guidelines, with all studies exceeding the 2024 EAU guidelines. The studies included in our systematic review revealed similar oncological outcomes after TA, among studies that followed the 2016 EAU guidelines and those that performed more frequent imaging, suggesting that more frequent imaging than the 2016 EAU guidelines may not lead to a survival benefit.

****Key Points**:**

***Question*** *Does more frequent follow-up CT imaging after tumor ablation for localized renal cell carcinoma improve oncological outcomes compared to European Association of Urology guideline recommendations?*

***Findings*** *89% of studies performed more frequent CT scans than the 2016 guidelines; recurrence was 7.7% with extra scans versus 12.3% with guideline adherence.*

***Clinical relevance*** *Current intensive imaging protocols may not improve patient outcomes, supporting potential reduction in follow-up imaging frequency to minimize radiation exposure and healthcare costs while maintaining adequate oncological surveillance.*

## Introduction

In the past two decades, tumor ablation (TA) has emerged as a widely used treatment modality for small renal masses, which are defined as contrast-enhancing solid or cystic lesions measuring up to 4 cm [1]. TA is less frequently employed for cT1b lesions (4-7 cm), as the oncological outcomes are less favorable [2, 3]. To date, various techniques are used for ablative treatment of renal tumors, including but not limited to cryoablation (CA), radiofrequency ablation (RFA), and microwave ablation (MWA). Although partial nephrectomy remains the reference standard for treatment of cT1 lesions, as stated in most international guidelines, retrospective series have demonstrated favorable oncological outcomes after TA [4–7].

As the TA assumes a more prominent role in the treatment of renal masses, adequate surveillance after treatment is of importance. Currently, no consensus exists regarding follow-up strategies after renal cell carcinoma (RCC) treatment. Depending on specific guideline recommendations, patients typically undergo multiple computed tomography (CT) scans over a 5-year period, sometimes extended to 10 years following TA, with the aim of timely detection of treatable recurrences. There are discrepancies in follow-up imaging recommendations among established guidelines, such as the European Association of Urology (EAU), the American Urological Association (AUA) and the National Comprehensive Cancer Network (NCCN) [8–10]. These recommendations are primarily based on the risk of recurrence, often using the Leibovich score for clear cell RCC and the TNM staging and International Society of Urological Pathology grading system for other subtypes [11].

Previous studies have shown that there is no evidence supporting that more frequent imaging leads to higher recurrence rates or higher survival rates [12]. A recent systematic review suggested that in patients surgically treated for localized RCC (radical or partial nephrectomy), more frequent imaging than recommended by the EAU guideline may not lead to a survival benefit [13]. However, this has not yet been investigated in the context of patients treated by TA, and no prior studies have evaluated the 2024 EAU RCC guidelines, underscoring the rationale for this systematic review. In addition, potential disadvantages of CT scans include higher healthcare costs, reduced availability of staff and resources, and greater cumulative radiation exposure for patients. Moreover, additional investigations may contribute to heightened anxiety among patients [14–16]. This systematic review, therefore, aims to assess whether imaging according to the 2016 and 2024 EAU RCC guidelines leads to different oncological outcomes compared to more frequent imaging Table 1 illustrates the imaging frequency proposed by the EAU 2016, EAU 2024, AUA and NCCN RCC guidelines.

**Table 1:** Follow-up schedules proposed by different guidelines for RCC after tumor ablation

Months post-ablative therapy										
	**3**	**6**	**12** **1 year**	**18**	**24** **2 years**	**30**	**36**	**48**	**60** **5 years**	**> 60**
EAU 2024 [1]		CT		CT		CT		> 3 yrs CT once every 2 yrs	^a^	
EAU 2016		CT	CT		CT		CT	CT	CT	CT once every 2 yrs up to 10 yrs
AUA 2021 [2]		CT	CT		CT		CT	CT	CT	Every 2 yrs up to 10 yrs
NCCN 2024 [3]	CT/MRI/CEUS	CT/MRI/CEUS	CT/MRI/CEUS		CT/MRI/CEUS		CT/MRI/CEUS	CT/MRI/CEUS	CT/MRI/CEUS	

*EAU* European Association of Urology, *AUA* American Urological Association, *NCCN* National Comprehensive Cancer Network, *CT* computed tomography, *MRI* magnetic resonance imaging, *US* ultrasound, *yr* year

^a^ Consider terminating follow-up for low-risk patients after 3 years of counselling based on assessment of comorbidities, age, life expectancy, and patient wishes

## Methods

### Evidence acquisition

#### Search strategy

This systematic review was conducted according to the Preferred Reporting of Items for Systematic Reviews and Meta-Analysis (PRISMA) guidelines [17]. The protocol was registered in the International Prospective Register of Systematic Reviews (PROSPERO) database (registration number CRD42025629702). The literature search was conducted by a medical librarian (F.J.) covering PubMed/MEDLINE and EMBASE (OVID) databases to identify relevant studies published up to June 6th, 2025. Retrieved references were deduplicated using a software tool (DedupEndNote [18] (Version 1.0.0)). The search strategy included the following terms: renal cell carcinoma, (tumor) ablation, ablative therapies, survival, and recurrence. The complete search strategy is available in the supplementary material (Appendix 1).

The following PICO (patient, intervention, comparison, and outcome) was developed for the search strategy:

*Population:* patients with cT1 RCC (all subtypes) who underwent tumor ablation (CA, RFA, MWA)

*Intervention:* studies with a higher frequency of CT chest/abdomen than recommended by the 2016 and 2024 EAU guidelines

*Control:* studies with equal frequency of CT chest/abdomen as recommended by the 2016 and 2024 EAU guidelines

*Outcome:* (time to) recurrence, survival data (overall survival (OS), cancer-specific survival (CSS), recurrence-free survival (RFS)/disease-free survival (DFS))

#### Study selection

In this review, retrospective and prospective cohort studies with patients aged ≥ 18 years who underwent TA (CA, RFA, MWA) as treatment for nonmetastatic cT1 RCC, that reported a follow-up schedule using CT of the abdomen/chest with a minimum follow-up duration of 2 years were eligible for inclusion. Notably, not all tumors were pathologically confirmed as RCC. Studies that did not report (time to) recurrence (local and distant metastases) or survival data (OS, CSS, or RFS/DFS) were excluded. Cohorts with < 20 patients were excluded. We selected this cut-off to improve the reliability of the reported outcomes and to minimize the impact of statistical fluctuations in small sample sizes.

Case reports, letters, editorials, congress abstracts and non-English studies were excluded. Articles published before 2003 were omitted due to outdated relevance regarding the introduction of TA in RCC. When multiple studies used the same cohort and outcome, the most recent were included; if outcomes were different, all outcomes were analyzed separately. The reference lists of included studies were screened for additional articles. Two reviewers (M.R. and L.B.) independently selected articles based on pre-defined criteria: disagreements were solved with a third reviewer (M.H.). Screening was conducted by using Rayyan [19] (Rayyan), an online tool for systematic reviews.

#### Data extraction

Data extraction was done by two authors (M.R. and L.B.). Potential queries were resolved by consulting a third party (M.H.). Baseline characteristics, follow-up schedules, recurrence and survival data were recorded. Technical aspects such as scan parameters, contrast protocols or modality-specific differences were not extracted. If studies also described results for other forms of treatment besides TA, for example, partial or radical nephrectomy, only the data of the patients who underwent TA as treatment were extracted. The imaging frequency was compared to the 2016 and 2024 EAU guidelines recommendations.

#### Outcome measurements

The outcomes of interest were (time to) recurrence of disease (local recurrences and distant metastases) and survival data, which included RFS/DFS, CSS or OS. If available, the 95%-confidence intervals were extracted for the RFS/DFS, CSS and OS. The data was not extracted from the Kaplan–Meier plots, only from tables and text. Additionally, a Forest plot was generated from the available 95% confidence intervals. This forest plot only displays the outcomes of the individual studies; no pooled effect was calculated.

#### Data synthesis

Baseline characteristics were summarized using descriptive statistics. Patients with benign histology were excluded from oncological outcomes. Sample-size weighted averages of variables such as age, follow-up duration, recurrence rates, and 5-year OS were calculated to provide a pooled descriptive summary across the included studies. Pooled summaries were also calculated separately for studies following the 2016 and 2024 EAU guidelines, and for those with more frequent imaging, but only if each group contained more than three studies. These summaries were compared. All analyses were conducted using R Studio (version 2025.05). Other findings were summarized with a narrative synthesis.

#### Risk of bias assessment

Three checklists were used to evaluate the methodological quality of the included studies; all checklists were specifically selected for the type of studies. The checklist commonly employed by the EAU for single-arm studies was used for case series that describe a specific type of TA [20]. The Robins-I tool was used for non-randomized studies comparing two or more interventions [21]. The Risk of Bias (RoB 2) tool developed by Cochrane was used for randomized studies [22]. All articles were assessed by the two independent reviewers (M.R. and L.B.). Disagreement was resolved by a third reviewer (M.H.).

## Evidence synthesis

### Literature search

**Fig. 1:**
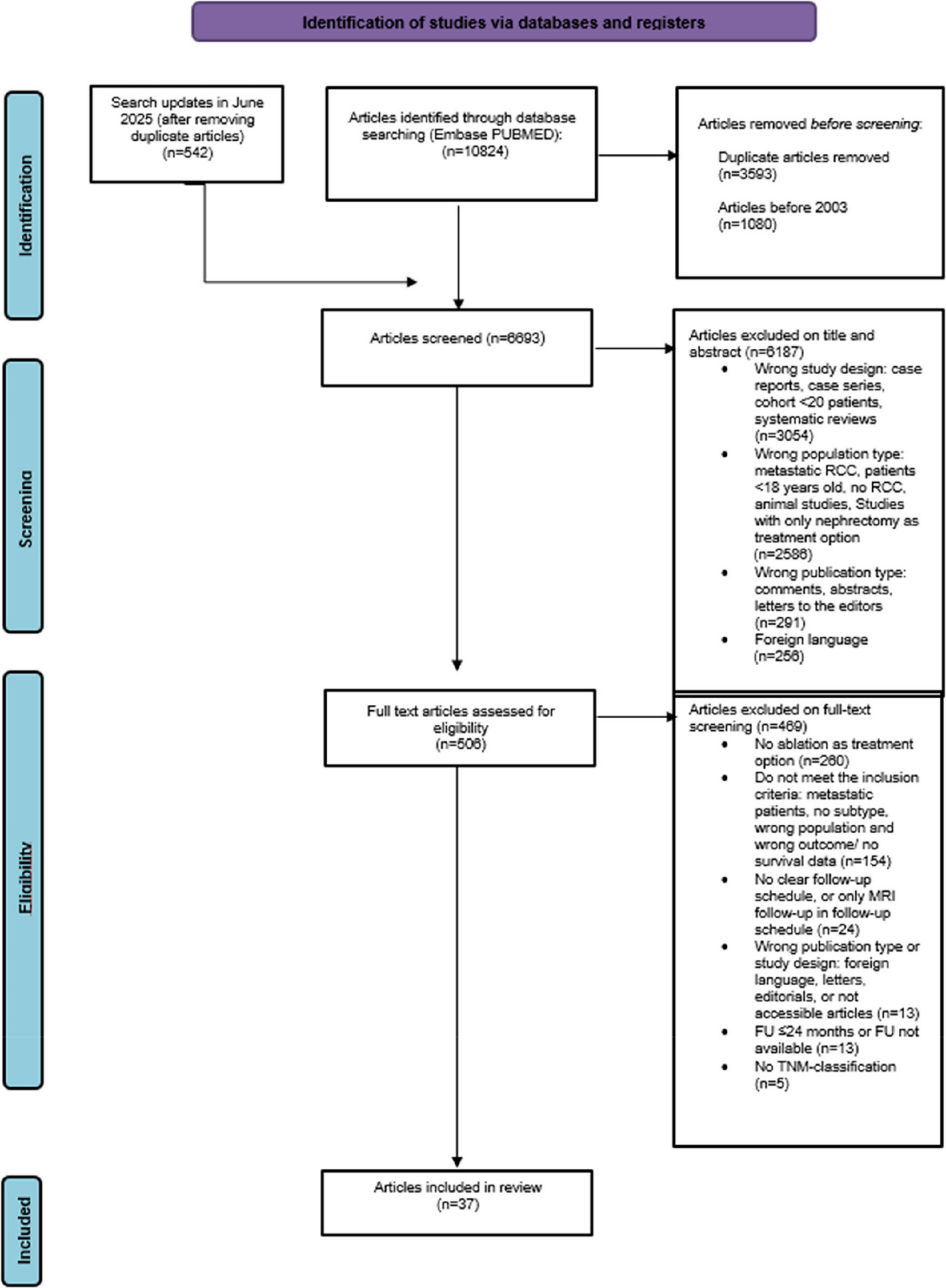
Flowchart of the in-and exclusion criteria

The literature search identified 7376 articles after removal of duplicates and those published before 2003. After exclusion of articles based on title and abstract (*n* = 6870), the remaining 506 articles were assessed for eligibility based on full text. In total, 37 articles were included. A flowchart of the inclusion process is shown in Fig. 1.

### Data extraction

#### Baseline characteristics

Among the 37 included studies, a total of 6067 tumors were described, of which 76.9% had pathology-proven RCC. All tumors were classified as cT1, with 5763 classified as cT1a (95.0%) and 304 as cT1b (5.0%). A total of 16 studies reported CA as the only treatment, 12 studies focused exclusively on RFA, five studies included multiple TA techniques, and four studies investigated only MWA. Overall, there were 34 retrospective cohort studies, two prospective studies [23, 24], and one randomized controlled trial (RCT) [25]. The weighted mean of median follow-up was 46.5 months. Table 2. presents the baseline characteristics.

**Table 2:** Baseline characteristics

Author, year	Study design	*N*	Age, mean (or median noted)	TNM	RCC Proven (%)	Tumor subtype	Type of intervention(s)	First CT scan	Imaging frequency compared to 2024 EAU	Imaging frequency compared to 2016 EAU
Aikawa, 2023 [37]	Retrospective cohort	29	*n* = 17 ≤ 80 yrs *n* = 12 > 80 yrs	cT1b	14 (48%)	CcRCC: 13, pRCC: 1	PCA	1 month	>	>
Andrews, 2019 [38]	Retrospective cohort	PCA 187, RFA 180	71 (65–79) PCA 72 (64–78) RFA	RFA cT1a 180 PCA cT1a 187 cT1b 52	PCA 108 (58%), RFA 73 (41%)	PCA: CcRCC: 73, pRCC 20, ChRCC 1, Other^a^ 14, RFA: CcRCC: 38, pRCC: 20 ChRCC: 3 Non-RCC: 12	PCA, RFA	3 months	>	>
Balageas, 2013 [29]	Retrospective cohort	62 (71 tumors)	74 (20–87)	cT1a	62 (100%)	CcRCC: 30, pRCC: 12, chRCC: 3, Non-RCC: 17	RFA	2–3 months	>	>
Beemster, 2011 [23]	Prospective cohort	92 (100 tumors)	69 (39–91)	cT1a	51 (54%)	CcRCC: 51	LCA	3 months	>	>
Bersang, 2021 [39]	Retrospective cohort	118	66	cT1a 124	67 (59%)	CcRCC: 50, pRCC: 15, Non-RCC: 2	RFA	3 months	>	>
Best, 2012 [26]	Retrospective cohort	159	63	cT1a 159	150 (94%)	RCC: 108, Non-RCC: 33	RFA	6 weeks	>	=
Bhagavatula, 2020 [40]	Retrospective cohort	307	68	cT1a 282, cT1b 25	307 (100%)	CcRCC: 125, pRCC: 65, chRCC: 4, Non-RCC: 113	PCA	3 months	>	>
Bianchi, 2021 [27]	Retrospective cohort	RFA 68, PCA 83	RFA 77, PCA 71	cT1a 137, cT1b 14	PCA 65 (78.3%) RFA 40 (58.8%)	PCA: CcRCC: 46, pRCC: 14, chRCC: 5, Non-RCC: 18 RFA: CcRCC: 21, pRCC: 16, chRCC: 3, Non-RCC: 28	PCA, RFA	6 months	>	=
Chan, 2022 [41]	Retrospective cohort	CA 103; RFA 100	73 (median)	cT1a 159 cT1b 44	T1a CA: 72 (100%), RFA: 87 (100%) T1b CA: 31 (100%), RFA: 13 (100%)	CA: CcRCC:45, pRCC: 6, chRCC: 19 EosRCC: 2 RFA: CcRCC 71, pRCC 5, ChRCC: 5 EosRCC: 6,	CA, RFA	1 month	>	>
Chang, 2015 [42] *After matching*	Retrospective cohort	45	53	cT1a	45 (100%)	CcRCC: 40, pRCC: 2, chRCC: 2, Non-RCC: 1	RFA	7 days	>	>
Chung, 2022 [30]	Retrospective cohort	39	62	cT1a	39 (100%)	CcRCC: 28, pRCC: 6, chrRCC: 5	RFA	1 month	>	>
Dreyfruss, 2019 [43]	Retrospective cohort	MWA 178, CA 78	67 (median)	cT1a 215, cT1b 41	256 (100%)	CcRCC: 190, pRCC: 45, chRCC: 10, Non-RCC: 11	MWA, PCA	3 months	>	>
Guan, 2012 [25]	Randomized study	48	46	cT1a	30 (63%)	CcRCC 27, pRCC 3	MWA	Within 24 hr	>	>
Guo, 2021 [31]	Retrospective cohort	106	69	cT1a	106 (100%)	CcRCC: 75, pRCC: 28, chRCC: 3	MWA	1 month	>	>
Haddad, 2018 [32]	Retrospective cohort	173	70 (median)	cT1a	173 (100%)	CcRCC: 130, pRCC: 43	PCA	Within 24 hr months	>	>
Johnson, 2014 [44]	Retrospective cohort	144	60	cT1a	109 (76%)	109:RCC	LCA	3 months	>	>
Karam, 2013 [45]	Retrospective cohort	150	69	cT1a	108 (72.1%)	CcRCC: 76, pRCC: 13, chRCC: 1, Non-RCC: 18	RFA	1 month	>	>
Lorber, 2014 [24]	Prospective cohort	50 (53 tumors)	68	cT1a 50, cT1b 3	53 (100%)	CcRCC: 38, pRCC: 12, chRCC: 1, Non-RCC: 2	RFA	1 month	>	=
Malcom, 2009 [46]	Retrospective cohort	66	67	cT1a	41 (62%)	41: RCC	LCA	1 month	>	>
Moulin, 2023 [47]	Retrospective cohort	25 (26 tumors)	65	cT1a	25 (100%)	CcRCC: 18, pRCC: 5, chRC: 1, Non-RCC: 2	PCA	1 month	>	>
Nielsen, 2017 [48]	Retrospective cohort	808	67	cT1a 808	589 (73%)	CcRCC: 364, pRCC: 92, chRCC: 43, Non-RCC: 90	LCA	3 months	>	>
Park, 2006 [49]	Retrospective cohort	78 (94 tumors)	64	cT1a 94	64 (77%)	RCC: 64	RFA	6 weeks	>	>
Park, 2019 [36]	Retrospective cohort	62	58	cT1a	62 (100%)	CcRCC: 49, pRCC: 3, chRCC: 6, Non-RCC: 4	RFA	1 month	>	>
Pedraza, 2023 [50]	Retrospective cohort	111	65	cT1a 109, cT1b 2	61 (56%)	CcRCC: 32, pRCC: 20, chRCC: 9	RFA	6 month	>	>
Piasentin, 2022 [51]	Retrospective cohort	315 (340 tumors)	75	cT1a	202 (67%)	RCC: 202	PCA	3 months	>	>
Pickersgill, 2020 [4]	Retrospective cohort	308 (328 tumors)	67	cT1a 296, ct1b 32	141 (43%)	CcRCC: 100, pRCC: 25, chRCC: 1, Non-RCC: 15	PCA	3 months	>	>
Psutka, 2013 [5]	Retrospective cohort	274	73	cT1a 143, cT1b 42	185 (100%)	CcRCC: 97, pRCC: 4, chRCC: 33, Non-RCC: 51	RFA	1 month	>	>
Ramirez, 2014 [34]	Retrospective cohort	79 (111 tumors)	64	cT1a	61 (77%)	RCC: 61	RFA	6 weeks	>	>
Shapiro, 2020 [28]	Retrospective cohort	40	69	cT1b	40 (100%)	CcRCC: 35, pRCC: 3, chRCC: 1, Non-RCC: 1	MWA	6 months	>	=
Stern, 2007 [35]	Retrospective cohort	40	61	cT1a	30 (75%)	CcRCC: 24, pRCC: 5, chRCC: 1, Non-RCC: 10	RFA	6 weeks	>	>
Sun, 2024 [57]	Retrospective cohort	257	70.5	cT1a	170 (66.1%)	CcRCC 107, pRCC 51, ChrRCC 1, Non-RCC: 1	PCA/MWA	4 months	>	>
Tanagho, 2013 [52]	Retrospective cohort	62	67	cT1a	35(52%)	CcRCC 16, pRCC 6, chRCC 1, Non-RCC: 39	LCA	Within 24 h	>	>
Umari, 2022 [53]	Retrospective cohort	59	71	cT1a	31 (53%)	CcRCC: 31	PCA	Within 1 month	>	>
Yamanoi, 2024 [54]	Retrospective cohort	108	70	cT1a 100, cT1b 8	108 (100%)	CcRCC: 90, Non-RCC: 18	PCA	1 month	>	>
Yanagisawa, 2020 [55] *After matching*	Retrospective cohort	90	69	cT1a 77, cT1b 13	65 (72%)	CcRCC: 60, pRCC: 3, chRCC: 2	PCA	1 month	>	>
Yu, 2020 [6] *After matching*	Retrospective cohort	185	63	cT1a	186 (100%)	CcRCC:174, pRCC: 5, chrRCC: 6	MWA	1 month	>	>
Zangiacomo, 2021 [56]	Retrospective cohort	85	63 (median)	cT1a	85 (100%)	CcRCC: 35, pRCC: 20, chRCC: 28, Non-RCC: 2	PCA	3 months	>	>

*RCT* randomized controlled trial, *PN* Partial nephrectomy, *FU* follow-up, *HR* high-risk, *IR* intermediate risk, *LR* low-risk, *CcRCC* clear cell, *pRCC* papillary, *chRCC* Chromophobe, *Trans* translocation, *Uncl* unclassified, *Cdc* collecting duct, *Eos* Eosinophilic, *sRCC* sarcomatoid RCC, *NR* not reported, *EAU* European Association of Urology, *LCA* laparoscopic cryoablation, *MWA* microwave ablation, *PCA* percutaneous cryoablation, *RCC* renal cell carcinoma, *RFA* radiofrequency ablation

^a^ Non-RCC was classified as benign tumors, oncocytomas, not diagnostic tumors or not specified tumors

#### Outcomes

##### *Imaging frequency compared to 2016 and 2024 EAU*

Of the 37 articles reviewed, four studies (10.8%) [24, 26–28] adhered to the 2016 EAU guidelines, while 33 employed a higher imaging frequency (89.2%). Notably, none of the included studies followed the imaging recommendations of the EAU 2024 guidelines, with all employing a higher imaging frequency. There were no studies that performed less imaging than recommended by the 2016 or 2024 EAU guidelines.

##### *First CT-scan after tumor ablation*

In nine studies [25, 29–36], a CT scan was performed after the treatment with the explicit purpose of evaluating the technical success of the procedure. The timing of these scans ranged from 24 h to 12 weeks post-treatment. In the remaining studies, where a scan to evaluate technical success was not specifically mentioned, the timing of the first CT scan varied between 6 weeks and 6 months post-treatment, as displayed in Table 2.

##### *Recurrences and time to recurrence*

The weighted analyses showed an overall recurrence rate of 8% (SD 2.43). The studies with more CT scans during follow-up than the 2016 EAU guidelines noted a mean recurrence rate of 7.7% [4–6, 23, 25, 28–32, 34, 36–57]. The studies that adhered to the 2016 EAU guidelines noted a mean recurrence of 12.3% (*p* = 0.19) [24, 26, 35].

#### *cT1a*

Of the 26 studies that included recurrence data of cT1a patients, recurrences ranged from 0 to 23.0% [6, 23, 25,26, 29–32, 34–36, 38, 39, 41, 42, 44–49, 51–53, 56, 57]. The studies by Chung et al and Park et al both found no recurrences after RFA at a median follow-up time of 60 months [30, 36]. Median time to recurrence was reported in 11 studies and ranged from 6 to 40 months [23, 31, 32, 34, 39, 44, 46, 47, 52, 56, 57]. One study followed the 2016 EAU guidelines and reported a recurrence rate of 5.3% at a median follow-up time of 54 months [26].

#### *cT1a and cT1b*

Of the nine articles that combined cT1a and cT1b patients, recurrences ranged from 4.6%–15.0% [4, 5, 24, 27, 40, 43, 50, 54, 55]. Median time to recurrence was reported in four studies and ranged from 15.1–32.0 months [4, 5, 24, 40]. Two studies adhered to the EAU 2016 guidelines, of which one noted a recurrence rate of 9.4% with a median time to recurrence of 32.0 months [24, 27].

#### *cT1b*

Among the two studies that included only cT1b patients, Aikawa et al noted recurrences in 57.0% and Shapiro et al in 5.0% of patients at a median follow-up time of 43 and 34 months, respectively. Neither study reported the time to recurrence. Shapiro et al adhered to the EAU guidelines, whereas Aikawa had a higher imaging frequency [28, 37].

##### *Survival*

All studies reported OS, CSS, RFS/DFS or a combination of these. The weighted analyses showed an overall 5-year survival of 82.9%.

#### *cT1a*

**Fig. 2:**
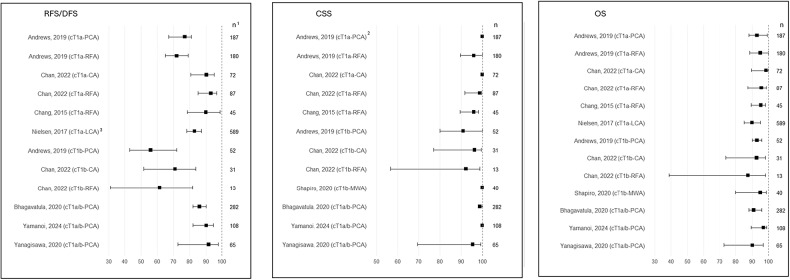
Forest plot of oncological outcomes (RFS/DFS, CSS, OS) by studies. ^1^ No pooled effect was calculated; the forest plots display the outcomes of individual studies. The RCC proven (n) is shown next to each point to provide context on the study size (number of proven RCC; see Table 3. When the number of proven RCC cases for a specific outcome was not reported, the total number of patients in that study was used), but it does not indicate statistical weighting. ^2^ For studies reporting exact values without a range (e.g., 100–100), the point estimate is plotted without a confidence interval. ^3^ In the text, a 95% CI of 95.2–98.4 was reported for a 5-year DFS estimate of 90.4. This value does not fall within the confidence interval. Upon reviewing the Kaplan–Meier plot, the 95% CI appeared to be around 85–95. CA, cryoablation; LCA, laparoscopic cryoablation; PCA, percutaneous cryoablation; RFA, radiofrequency ablation. CSS, Cancer-specific survival; DFS, Disease-free survival; OS, Overall survival; RFS, Recurrence-free survival

The 5-year RFS/DFS rate was reported in 12 studies, ranging from 86.0%–100% [26, 30, 32, 34, 36, 38, 39, 41, 42, 44, 48, 56]. Ten studies reported a 5-year CSS ranging from 96.0%–100% [29, 34, 36, 38, 41, 42, 44, 53, 56, 57]. The 5-year OS rate was shown in ten studies and ranged from 71%–98% [6, 34, 36, 38, 41, 42, 44, 48, 53, 57]. The one study that adhered to the 2016 EAU guideline did not report 5-year OS [26]. The 5-year survival rates with 95.0% confidence intervals are shown in Fig. 2.

#### *cT1a and cT1b*

The 5-year RFS/DFS rate was reported in six studies, ranging from 77.0%–97.0% [5, 24, 27, 40, 43, 54]. Six studies reported a 5-year CSS ranging from 96.0%–100% [5, 24, 40, 43, 54, 55]. The 5-year OS rate was shown in four studies and ranged from 86.0%–98.0% [24, 40, 54, 55]. Of the two studies that adhered to the 2016 EAU guidelines [24, 27], only one reported a 5-year OS rate of 98.0% [24].

#### *cT1b*

Four studies reported survival analyses specifically for cT1b patients [28, 37, 38, 41]. The 5-year RFS/DFS ranged from 77.0%–95.0% [28, 38, 41], the 5-year CSS was between 91.0%–100% [28, 37, 38, 41], and the 5-year OS was between 56.0%–84.0% [37, 38, 41]. Shapiro et al, the only study that followed the 2016 EAU guidelines, did not report an OS, though it had a high 5-year CSS of 100% [28]. The recurrence and survival data can be found in Table 3.

**Table 3:** Number of recurrences and survival analyses

Author	RCC proven	Length of FU (months) median (IQR or range)	Overall recurrences	Local recurrences	Distant recurrences	Time to recurrence (months)	RFS/DFS (95% CI)^a^	CSS (95% CI)	Overall survival (95% CI)	Imaging frequency compared to 2024 EAU	Imaging frequency compared to 2016 EAU
Aikawa, 2023 [37]	14	43 months	8 (57)	4	4	NR	NR	5 yr. 97%	5 yr. 84%	>	>
Andrews, 2019 [38]	PCA 108, RFA 73	PCA 6.3 yr RFA 7.5 yr	22 (12)	PCA: cT1a: 6 (27)cT1b: 3 (14) RFA: cT1a: 6 (27)	PCA: cT1a: 1 (4.5) cT1b: 2 (9.0) RFA: cT1a: 4 (18)	NR	RFS 5 yr. PCA: cT1a: 93% (88.0–99.3), cT1b: 93% (90–96), RFA: cT1a: 95% (88.6–100)	5 yr. PCA: cT1a: 100%, (100–100) cT1b: 91% (80–100), RFA: cT1a: 96% (89.7–100)	5 yr. PCA: cT1a: 77% (67–81), cT1b: 56% (43–722), RFA: cT1a: 72% (65–79)	>	>
Balageas, 2013 [29]	62	36.5 months	9 (15)	5	4	NR	< 4 cm 100% > 4 cm 57.1%^b^	5 yr. 97%	NR	>	>
Beemster, 2011 [23]	51	30.2 months (mean)	4 (7.8)	4	0	23 months	3 yr. 92% (76.3–97.3)	3 yr. 100% (100-100)	3 yr. 86% (71.2–93.6)	>	>
Bersang, 2021 [39]	67	5 yr	6 (9.0)	6	0	4.9 yr (1.5-8.4)	5 yr. 93% 10 yr. 84%	10 yr. 96%	NR	>	>
Best, 2012 [26]	150	54 months (1.5-120)	8 (5.3)	6	2	NR	3 yr. 92%, 5 yr. 91%	NR	NR	>	=
Bhagavatula, 2020 [40]	307	95 months (8-219)	16 (5.2)	13	3	21 months	5 yr: 91% (88–96), 10 yr: 88% (83–93)	5 yr: 99% (98-100), 10 yr: 99% (98–100)	5 yr: 86% (82–90), 10 yr: 78% (73–84)	>	>
Bianchi, 2021 [27]	105	61 months (33-93)	RFA 11 (23.9) CA 12 (16.9)	NR	NR	NR	3 yr. PCA: 90% RFA: 84% 5 yr. PCA: 88% RFA: 79%	NR	NR	>	=
Chan, 2022 [41]	203	CA 75.6 months (66.8-86.5), RFA 106 (61.2-135.1)	cT1a: 9 (5.7) CA 2; RFA 7 cT1b: 6 (11) CA 4; RFA 2	11 cT1a: CA 2; RFA 5 cT1b: CA 3; RFA 1	7 cT1a: CA 0; RFA 2 cT1b: CA 1; RFA 1	NR	CA T1a 5 yr: 98.5% (89.7–99.8) 10 yr: 92.3% (66.7–98.5) T1b 5 yr: 92.8% (73.9–98.1) 10 yr: 86.4% (61.7-95.6), RFA T1a 5 yr: 95.7% (87.3–98.6) 10 yr: 91.4% (80.0–96.4), T1b 5 yr: 87.5% (38.7–98.1) 10 yr: 87.5%) (38.7–98.1)	CA T1a 5 yr: 100% (N/A) 10 yr: 100% (N/A) T1b 5 yr: 96.4% (77.2–99.5) 10 yr: 96.4% (77.2-99.5) RFA T1a 5 yr: 98.8% (91.8–99.8) 10 yr: 98.8% (91.8–99.8), T1b 5 yr: 92.3% (56.6–98.9) 10 yr: 92.3% (56.6–98.9)	CA T1a 5 yr: 90.3%, (80.7–95.2) 10 yr: 73.9% (56.4–85.2), T1b 5 yr: 71% (51.6–83.7) 10 yr: 43.5% (24.5–61.9), RFA T1a 5 yr: 93% (85.0–96.8) 10 yr: 89% (79.9–94.1) T1b 5 yr 61.5% (30.8–81.8) 10 yr 52.8% (23.4–75.5)	>	>
Chang, 2015 [42]	45	38 months	2 (4.4)	2	NR	NR	5 yr. 95.4 (89.3–98.1)	5 yr. 96% (89.5–98.1)	5 yr. 90% (78.6–98.8)	>	>
Chung, 2022 [30]	39	60 months	0	0	0	NR	5 yr. 97%	NR	NR	>	>
Dreyfruss, 2019 [43]	256	31.6 months	23 (8.9)	16	7	NR	5 yr. 89%	5 yr. 99%	NR	>	>
Guan, 2012 [25]	30	32 months	2 (6.7)	NR	NR	NR	3 yr. RFS 90% (65.3–97.6) DFS 100%	NR	NR	>	>
Guo, 2021 [31]	106	24 months	6 (5.7)	6	0	20 months	PFS 3 yr. 90.6%	NR	3 yr. 95%	>	>
Haddad, 2018 [32]	173	26 months	6 (3.5)	5	1	24 months	5 yr. ccRCC 88% pRCC 100%	NR	NR	>	>
Johnson, 2014 [44]	109	97.9 months	6 (5.5)	5	1	40 months	5 yr. 94% 10 yr. 87%	5 yr. 100% 10 yr. 98%	5 yr. 88% 10 yr. 71%	>	>
Karam, 2013 [45]	108	25.0 months	4 (3.7)	4	0	NR	NR	100% at 39 months	NR	>	>
Lorber, 2014 [24]	53	65.6 months	5 (9.4)	4	1	32 months	5 yr. 93%	5 yr. 100%	5 yr. 98%, 10 yr. 93%	>	=
Malcom, 2009 [46]	41	25.1 months	7 (17)	7	NR	10.2	97% at 30 months	100% at 30 months	100% at 30 months	>	>
Moulin, 2023 [47]	25	795 days	2 (8)	NR	2	200 days, 400 days	92%	NR	NR	>	>
Nielsen, 2017 [48]	589	36 months	20 (3.4)	16	4	NR	5. yr 90% ( ≈ 85–95)^c^ 10 yr. 80% (67.2–98.4)	NR	5 yr. 83% (78.2–87.2) 10 yr. 64% (44.5–78.7)	>	>
Park, 2006 [49]	64	25 months (mean)	3 (4.7)	3	0	NR	97% at median FU	100% at median FU	96% at median FU	>	>
Park, 2019 [36]	62	60 months (mean)	0 (0)	0	0	NR	5 yr. 100%	5 yr. 100%	5 yr. 98%	>	>
Pedraza, 2023 [50]	61	38 months	4 (6.6)	4	0	NR	HR 0.9, 95% CI, 0.28–2.9, *p* = 0.86	NR	HR 1.2, 95% CI, 0.42–3.1, *p* = 0.78	>	>
Piasentin, 2022 [51]	202	27.9 months	15 (7.4)	15	0	NR	2 yr. 95%	NR	NR	>	>
Pickersgill, 2020 [4]	141	37.8 months (mean)	21 (15)	12	9	15.1–16.3 months	1 yr: T1a: 96% T1b: 69% 2 yr: T1a: 93% T1b: 61% 3 yr. T1a: 90% T1b: 61%	NR	1 yr: T1a: 98% T1b: 96% 2 yr: T1a: 94% T1b: 80% 3 yr. T1a: 88% T1b: 68%	>	>
Psutka, 2013 [5]	185	6.4 yr	16 (8.6)	12	4	30 months	5 yr. 95% (local), 5 yr. 99% (metastasis)	5 yr. 99%	5 yr. 73%	>	>
Ramirez, 2014 [34]	61	59 months	5 (8.2)	5	0	39 months	5 yr. 93%	5 yr. 100%	5 yr. 72%	>	>
Shapiro, 2020 [28]	40	34 months	2 (5.0)	2	0	NR	5 yr. 95% (79.8-98.6)	5 yr. 100% (N/A)	NR	>	=
Stern, 2007 [35]	30	30 months (mean)	4 (13)	4	0	NR	3 yr. RFS 91%, DFS 100%	NR	NR	>	>
Sun, 2024 [57]	170	29.5 months	7 (4.1)	5	2	9.6–15.6 months	NR	MWA 100%, CA 98.2%	MWA 91.2%, CA 70.8%	>	>
Tanagho, 2013 [52]	35	76 months (mean)	14 (8)	5	0	28 months	6 yr. 80%	NR	NR	>	>
Umari, 2022 [53]	31	33.3 months	7 (23)	6	1	NR	NR	5 yr. 98%	5 yr. 97%	>	>
Yamanoi, 2024 [54]	108	61 months	5 (4.6)	3	2	NR	5 yr. LRFS 97% (89.4–98.9) MFS 98% (91.9–99.5)	5 yr. 100%	5 yr. 90.2% (81.9–94.8)	>	>
Yanagisawa, 2020 [55] *After matching*	65	26.5 months	8 (12)	4 local recurrences, 3 tumor residues	1	NR	5 yr. LRFS 90.2 (72.7–96.8)	5 yr. 95.5% (69.5–99.3)	5 yr. 91.7% (72.7–97.7)	>	>
Yu, 2020 [6] *After matching*	186	42 months	23 (12)	7	16	NR	NR	NR	1 yr. 98% 3 yr. 94% 5 yr. 86%	>	>
Zangiacomo, 2021 [56]	85	56 months	4 (4.7)	4	NR	36 months	1 yr. 98.8% LTPFS 5 yr. 93%	100%	1 and 3 yr.100% 5 yr. 98%	>	>

*LTPFS* local tumor progression-free survival, *CSS* Cancer-specific survival, *DFS* Disease-free survival, *EAU* European Association of Urology, *LCA* laparoscopic cryoablation, MWA microwave ablation, *NR* not reported, *PCA* percutaneous cryoablation, *RCC* renal cell carcinoma, *RFA* radiofrequency ablation, *RFS* recurrence-free survival

^a^ If the 95%-CI was reported

^b^ Not clear at what time the RFS/DFS was measured

^c^ In the text, a 95% CI of 95.2–98.4 was reported for a 5-year DFS estimate of 90.4. This value does not fall within the confidence interval. Upon reviewing the Kaplan–Meier plot, the 95% CI appeared to be around 85–95

##### *Risk of bias and quality assessment*

The risk of bias was moderate to high among single-arm studies and high among non-randomized studies comparing two interventions. There was one randomized study that had a low risk (LR) of bias. The full presentation of the risk of bias data can be found in Table 4.

**Table 4:** Overall risk of bias included all included studies (green = low risk, orange = moderate risk, red = serious risk, dark brown = critical risk)

Author (year)	Overall risk of bias	Type of risk of bias tool
Aikawa, 2023 [37]		ROBINS-I tool
Andrews, 2019 [38]		ROBINS-I tool
Balageas, 2013 [29]		RoB: Case series
Beemster, 2011 [23]		RoB: Case series
Bersang, 2021 [39]		RoB: Case series
Best, 2012 [26]		RoB: Case series
Bhagavatula, 2020 [40]		RoB: Case series
Bianchi, 2021 [27]		ROBINS-I tool
Chan, 2022 [41]		ROBINS-I tool
Chang, 2015 [42]		ROBINS-I tool
Chung, 2022 [30]		ROBINS-I tool
Dreyfruss, 2019 [43]		ROBINS-I tool
Guan, 2012 [25]		ROB2 tool for randomized studies
Guo, 2021 [31]		RoB: Case series
Haddad, 2018 [32]		RoB: Case series
Johnson, 2014 [44]		RoB: Case series
Karam, 2013 [45]		RoB: Case series
Lorber, 2014 [24]		RoB: Case series
Malcolm, 2009 [46]		RoB: Case series
Moulin, 2023 [47]		RoB: Case series
Nielsen, 2017 [48]		RoB: Case series
Park, 2006 [49]		RoB: Case series
Park, 2019 [36]		ROBINS-I tool
Pedraza, 2023 [50]		RoB: Case series
Piasentin, 2022 [51]		RoB: Case series
Pickersgill, 2020 [4]		RoB: Case series
Psutka, 2013 [5]		RoB: Case series
Ramirez, 2014 [34]		RoB: Case series
Shapiro, 2020 [28]		ROBINS-I tool
Stern, 2007 [35]		ROBINS-I tool
Sun, 2024 [57]		ROBINS-I tool
Tanagho, 2013 [52]		RoB: Case series
Umari, 2022 [53]		ROBINS-I tool
Yamanoi, 2024 [54]		ROBINS-I tool
Yanagisawa, 2020 [55]		ROBINS-I tool
Yu, 2020 [6]		ROBINS-I tool
Zangiacomo, 2021 [56]		RoB: Case series

## Discussion

In our systematic review evaluating the use of CT scans during follow-up after TA, 10.8% of the studies adhered to the 2016 EAU guideline recommendations, while the remaining 89.2% employed a higher imaging frequency. All studies reported a higher imaging frequency than recommended by the updated 2024 EAU guidelines for low-risk patients, and the timing of the first CT scan varied among articles and did not depend on the type of ablation. The weighted analyses showed a recurrence rate in articles with a higher imaging frequency (7.7%) compared to those adhering to the 2016 guidelines (12.3%) (*p* = 0.19). We did not observe a relationship between imaging frequency and 5-year OS, as all studies that reported OS had a higher imaging frequency compared to the guidelines. Most studies exhibited a moderate to high risk of bias.

To date, radiological follow-up strategies after TA lack clarity and are not based on high-quality evidence. In fact, there is one randomized study that reports on the oncological outcomes after TA [25]. A Delphi consensus project in 2016 proposed several recommendations, such as that the first CT scan to check for residual disease should be done three months after TA. In the first year of follow-up, at least two imaging examinations were advised. Furthermore, the majority of the Delphi panel members recommended a 10-year follow-up period due to the absence of long-term data on the oncological outcomes after TA [58]. Despite the more risk-stratified follow-up recommendations in the 2024 EAU guidelines, none of the included studies adhered to these updated imaging protocols. All studies applied more frequent imaging than is currently advised. Therefore, this review does not provide direct outcome data from studies following the 2024 guidelines, but rather highlights a significant gap between guideline recommendations and clinical practice. This discrepancy underscores the need for evaluating long-term outcomes that reflect the 2024 EAU recommendations.

In our systematic review, we were unable to identify a specific pattern in the time interval between the first CT scan across ablation modalities. Again, the EAU guidelines make no distinction between ablative modalities in their follow-up recommendations. Notably, CA allows real-time assessment of technical success, while with MWA and RFA, this evaluation must be done after the procedure [59–61]. This difference could lead to differences in the timing of the first CT scan post-TA.

In terms of recurrences and survival, the studies that employed a higher scanning frequency reported similar recurrence and survival rates to those of the four studies that adhered to the 2016 EAU guidelines. In addition, the 10-year survival rate ranged between 92.0 and 98.0% [39–41, 44]. Despite the retrospective nature of the included studies, these high survival rates prompt the question of whether this follow-up duration is necessary. Additionally, a previous study by Dabestani et al indicated that the risk of recurrence is minimal for patients with low-risk disease [62].

Given these findings, consideration should also be given to the potential drawbacks of higher scanning frequency. The associated costs of imaging, use of possibly limited resources, and, in younger individuals, the cumulative radiation exposure, should also be recognized when considering these follow-ups [14, 63]. The study by Lobo et al compared different international guidelines concerning radiation exposure and the associated costs for patients treated with partial nephrectomy. This study demonstrated that more expensive protocols (using CT at higher frequencies) do not necessarily improve recurrence detection rates [64]. Another drawback of higher scanning frequency is the experience of increased anxiety in relation to follow-ups, especially in the time between the scan and receiving the results [15]. Additionally, another review by Wullaert et al examined the impact of a less intensive surveillance strategy on Health Related Quality of Life (HRQoL). Of the seven studies that specifically focused on reducing radiological follow-up, five of these found no significant differences in the HRQoL [16]. In summary, besides oncological outcomes, factors such as radiation exposure, costs and patient anxiety should be considered in follow-up schedules.

Our systematic review has several strengths. Firstly, this systematic review provides an objective and comprehensive overview of the available evidence, which summarizes the current use of CT follow-up after TA for cT1 RCC, by assessing recurrence rates and OS using a weighted analysis. Secondly, this review compares the most recent 2024 guidelines with the 2016 EAU guidelines, given that many of these studies were conducted before 2016 or between 2016 and 2024. Third, our review emphasizes the lack of adherence to the EAU guidelines in studies, and mainly the surplus of imaging performed during follow-up.

Nevertheless, this review has some limitations. First, a limitation is that we could not adjust for confounders such as tumor size, subtype and patient comorbidity due to the retrospective nature of the studies and their inherent lack of methodological description. Secondly, because most studies were retrospective, this results in a moderate to high risk of bias, with only one randomized study showing a LR of bias. The lack of high-quality studies on this topic limits us in drawing robust conclusions on the effect of performing more CT scans during follow-up compared to guideline recommendations. Finally, it should be noted that none of the included studies adhered to the 2024 follow-up recommendations, which may limit the applicability of our review. However, this systematic review clearly illustrates the current practice of a high imaging frequency during follow-up after ablation of renal tumors, which should prompt further research on the possible reduction of CT scans.

There was variation in the timing of the first CT scan reported in the included studies. Additionally, some studies specified that the first scan was to determine technical success, whereas others did not clearly state this in their methods. Further clarification in future studies regarding the rationale and timing of initial post-treatment imaging would be valuable. Furthermore, all tumors in this study were classified into a low-risk group, based on cTNM and histology [65]. Nonetheless, in some cases, there could have been upstaging to a pT3a lesion, thereby affecting the risk of recurrence and OS. Additionally, it is possible that some articles considered oncocytomas as malignant and included these in oncological outcomes, which may have skewed the survival outcomes more favorably [5, 26, 27, 35, 36, 42, 45, 47]. These factors could have contributed to an overestimation of the survival data. Finally, only one study adhering to the 2016 guidelines reported 5-year OS, preventing direct comparison with the studies that performed more frequent imaging.

Moreover, we recommend that future prospective studies should focus on reducing imaging frequency during follow-up and examine the effect on OS. A recent publication by Dabestani et al revealed the potential for urine glycosaminoglycan (GAGs) in the surveillance for recurrences. The GAGs were shown to have a high sensitivity (90.0%) and negative predictive value (97.0%) [66]. These developments are promising non-invasive techniques that could potentially substitute imaging in the future.

## Conclusion

This systematic review demonstrates that most of the studies used a higher imaging frequency during follow-up after TA than recommended by the 2016 and all studies compared to the 2024 EAU guidelines. Considering the low recurrence rates and high CSS found among the included studies, more imaging than recommended by the 2016 guidelines may not lead to improved survival. Unfortunately, evidence for the 2024 EAU guidelines is lacking, so no conclusion can be drawn yet regarding the potential benefit of intensified follow-up. Moreover, follow-up schedules with lower imaging frequency should be incorporated into prospective studies to determine the optimal follow-up schedule for future evidence-based guidelines.

## Supplementary information

Supplementary information

## **Abbreviations**

AUA: American Urology Association
CA: Cryoablation
CSS: Cancer-specific survival
DFS: Disease-free survival
EAU: European Association of Urology
HR: High risk
IR: Intermediate risk
LR: Low risk
MWA: Microwave ablation
NCCN: National Comprehensive Cancer Network
OS: Overall survival
RCC: Renal cell carcinoma
RFA: Radiofrequency ablation
RFS: Recurrence-free survival
TA: Tumor ablation
