# Supplementary material for: The use of computed tomography during follow-up after ablation of cT1 renal cell carcinoma: evidence for overuse
Source: Eur Radiol. 2026 Feb 6;36(7):5317–33. doi: 10.1007/s00330-026-12345-6 (PMC13282219; doi:10.1007/s00330-026-12345-6)
Supplement: Supplementary file 1 — Supplementary information [file 330_2026_12345_MOESM1_ESM.pdf]

The use of Computed Tomography during Follow-up After Ablation of cT1 Renal Cell Carcinoma: Evidence for Overuse
ELECTRONIC SUPPLEMENTARY MATERIAL

Appendices

Appendix 1. Full search strategy

Current radiological follow-up strategies in patients with localized renal cell carcinoma after primary treatment: a systematic review of the literature.

Search: Faridi Jamaludin, Amsterdam UMC location University of Amsterdam, Medical Library AMC, Meibergdreef 9, Amsterdam, The Netherlands

UPDATE SEARCH 19-11-2024 – 5-6-2025:

5-6-2025:

Databases:	Before deduplication	After deduplication*
PubMed, Embase (Ovid)		
Total	956	542

*Lobbestael, G. (2023). DedupEndNote (Version 1.0.0) [Computer software]. https://github.com/globbestael/DedupEndNote

PUBMED

187 results

("Carcinoma, Renal Cell"[Mesh] OR renal cell carcinom*[tiab] OR renal carcinom*[tiab] OR renal cancer*[tiab])

AND

("Surgical Procedures, Operative"[Mesh] OR nephrectom*[tiab] OR ablation*[tiab] OR ablative[tiab] OR surgical[tiab] OR surger*[tiab])

AND

("Survival Analysis"[Mesh] OR "Survival"[Mesh] OR "Recurrence"[Mesh] OR "Neoplasm Recurrence, Local"[Mesh] OR recurr*[tiab] OR surviv*[tiab])

AND

("Clinical Trial" [Publication Type] OR "Randomized Controlled Trial" [Publication Type] OR "Cohort Studies"[Mesh] OR "Controlled Clinical Trial" [Publication Type] OR random*[tiab] OR trial*[tiab] OR cohort[tiab] OR retrospectiv*[tiab] OR prospectiv*[tiab])

AND ("2024/11/19"[Date - Publication] : "3000/12/31"[Date - Publication])

EMBASE (OVID):

Database(s): Embase Classic+Embase 1947 to 2025 June 04
Search Strategy:

#	Searches	Results
1	renal cell tumor/ or exp renal cell carcinoma/	50286
2	(renal cell carcinom* or renal carcinom* or renal cancer*).ti,ab,kf.	93713
3	1 or 2	111188
4	surgery/ or exp abdominal surgery/ or exp cancer surgery/ or elective surgery/ or exp surgical approach/ or exp urologic surgery/	3125476
5	(nephrectom* or ablation* or ablative or surgical or surger*).ti,ab,kf.	3781953
6	4 or 5	5300355
7	exp survival analysis/ or exp survival/ or exp survival rate/	1671882
8	recurrent disease/	248471
9	tumor recurrence/	79289
10	cancer recurrence/	307879
11	(recurr* or surviv*).ti,ab,kf.	3383393
12	7 or 8 or 9 or 10 or 11	3845320
13	exp clinical trial/	2417026
14	cohort analysis/	1369951
15	(random* or trial* or cohort or retrospectiv* or prospectiv*).ti,ab,kf.	7232174
16	13 or 14 or 15	8197904
17	3 and 6 and 12 and 16	10533
18	limit 17 to conference abstracts	3919
19	17 not 18	6614
20	limit 19 to yr="2024 -Current"	769

UPDATE SEARCH 28-5-2024 t/m 19-11-2024:

19-11-2024:

Databases:	Before deduplication	After deduplication*
PubMed, Embase (Ovid)		
Total	637	288

*Lobbestael, G. (2023). DedupEndNote (Version 1.0.0) [Computer software]. https://github.com/globbestael/DedupEndNote

PUBMED

166 results

("Carcinoma, Renal Cell"[Mesh] OR renal cell carcinom*[tiab] OR renal carcinom*[tiab] OR renal cancer*[tiab])

AND

("Surgical Procedures, Operative"[Mesh] OR nephrectom*[tiab] OR ablation*[tiab] OR ablative[tiab] OR surgical[tiab] OR surger*[tiab])

AND

("Survival Analysis"[Mesh] OR "Survival"[Mesh] OR "Recurrence"[Mesh] OR "Neoplasm Recurrence, Local"[Mesh] OR recurr*[tiab] OR surviv*[tiab])

AND

("Clinical Trial" [Publication Type] OR "Randomized Controlled Trial" [Publication Type] OR "Cohort Studies"[Mesh] OR "Controlled Clinical Trial" [Publication Type] OR random*[tiab] OR trial*[tiab] OR cohort[tiab] OR retrospectiv*[tiab] OR prospectiv*[tiab])

AND ("2024/05/28"[Date - Publication] : "3000/12/31"[Date - Publication])

EMBASE (OVID):

Database(s): Embase Classic+Embase 1947 to 2024 November 18
Search Strategy:

#	Searches	Results
1	renal cell tumor/ or exp renal cell carcinoma/	44644
2	(renal cell carcinom* or renal carcinom* or renal cancer*).ti,ab,kf.	89727
3	1 or 2	105799
4	surgery/ or exp abdominal surgery/ or exp cancer surgery/ or elective surgery/ or exp surgical approach/ or exp urologic surgery/	2869911
5	(nephrectom* or ablation* or ablative or surgical or surger*).ti,ab,kf.	3573806
6	4 or 5	4966324
7	exp survival analysis/ or exp survival/ or exp survival rate/	1602685
8	recurrent disease/	236302
9	tumor recurrence/	77339
10	cancer recurrence/	283803
11	(recurr* or surviv*).ti,ab,kf.	3233012
12	7 or 8 or 9 or 10 or 11	3676683
13	exp clinical trial/	1983024
14	cohort analysis/	1245748
15	(random* or trial* or cohort or retrospectiv* or prospectiv*).ti,ab,kf.	6737407
16	13 or 14 or 15	7540064
17	3 and 6 and 12 and 16	9806
18	limit 17 to conference abstracts	3769
19	17 not 18	6037
20	limit 19 to yr="2024 -Current"	471

Appendix 2. The risk of bias tables

Table S1a. Risk of bias: case series (green = low risk, orange = moderate risk, red = high risk)

Author (year)	1.Was there an *a priori* protocol?	2.Was the total eligible population included and recruited consecutively?	3.Was outcome data complete for all participants and any missing data adequately explained/unlikely to be related to the outcome?	4.Were all pre-specified outcomes of interest and expected outcomes reported?	5.Were primary benefit and harm outcomes appropriately measured?	Overall risk of bias
Balageas, 2013						
Beemster, 2011						
Bersang, 2021						
Best, 2012						
Bhagavatula, 2020						
Guo, 2021						
Haddad, 2018						

Johnson, 2014						
Karam, 2013						
Lorber, 2014						
Malcolm, 2009						
Moulin, 2023						
Nielsen, 2017						
Park, 2006						
Pedraza, 2023						
Piasentin, 2022						
Pickersgill, 2020						
Psutka, 2013						
Ramirez, 2014						
Tanagho, 2013						

Zangiacomo, 2021						

Table S1b. Risk of bias: ROBINS-I tool (green = low risk, orange = moderate risk, red = serious risk, dark brown = critical risk)

**Author (year)**	**1. RoB due to confounding**	**2. RoB in classification of interventions**	**3. RoB in selection of participants into the study**	**4. RoB due to deviations from intended interventions**	**5. RoB due to missing data**	**6. RoB arising from measurement of the outcome**	**7. RoB in selection of the reported results**	**Overall risk**
Aikawa, 2023								
Andrews, 2019								
Bianchi, 2021								
Chan, 2022								
Chang, 2015 (RFA/part.nefr)								
Chung, 2022 (RFA/part.nefr)								
Dreyfruss, 2019								
Park, 2019								
Shapiro, 2020								
Stern, 2007								
Sun, 2024								
Umari, 2022								

Yamanoi, 2024								
Yanagisawa, 2020								
Yu, 2020								

*Table S1c***.** Risk of bias: ROB2 tool for randomized studies (green = low risk, orange = moderate risk, red = high risk)

**Author, year**	**1.Randomization**	**2. Deviations from the intended intervention**	**3. Missing data**	**4. Outcome measurement**	**5. Selection of reported results**	**Overall risk of bias**
Guan, 2012	+	+	-	+	+	+

Appendix 3. Weighted mean recurrence rates

Table S2a. Weighted mean recurrence rates across all included studies and subgroups

Number of studies included	Total participants	Weighted mean recurrence (%)	95% CI	Average participants per study	p-value
N= 37	5551	8%	(7.3-8.7)	150	-
	Subgroup analysis				
Followed the EAU 2016 guidelines N=4	400	12.3%	(9.1-15.5)	100	0.19[1]
More follow-up CTs than EAU 2016 guidelines N=33	5151	7.7%	(7.0-8.4)	156	

[1] P-value from two-sample test for proportions (two-tailed). No significant difference in recurrence rates was observed between the two subgroups (p ≈ 0.19).
